# A Dual Strategy of Breeding for Drought Tolerance and Introducing Drought-Tolerant, Underutilized Crops into Production Systems to Enhance Their Resilience to Water Deficiency

**DOI:** 10.3390/plants9101263

**Published:** 2020-09-24

**Authors:** Amparo Rosero, Leiter Granda, Jhon A. Berdugo-Cely, Olga Šamajová, Jozef Šamaj, Radim Cerkal

**Affiliations:** 1 Corporación Colombiana de Investigación Agropecuaria–AGROSAVIA, Centro de Investigación Turipaná, Km 13 vía Montería, 250047 Cereté, Colombia; jberdugo@agrosavia.co; 2 Department of Crop Science, Breeding and Plant Medicine, Mendel University in Brno, Zemedelska 1, 613 00 Brno, Czech Republic; leiter.granda@gmail.com (L.G.); radim.cerkal@mendelu.cz (R.C.); 3 Department of Cell Biology, Centre of the Region Haná for Biotechnological and Agricultural Research, Faculty of Science, Palacký University, Šlechtitelů 27, 783 71 Olomouc, Czech Republic; olga.samajova@upol.cz (O.Š.); jozef.samaj@upol.cz (J.Š.)

**Keywords:** crop diversity, drought tolerance, genetic approaches, neglected and underutilized species

## Abstract

Water scarcity is the primary constraint on crop productivity in arid and semiarid tropical areas suffering from climate alterations; in accordance, agricultural systems have to be optimized. Several concepts and strategies should be considered to improve crop yield and quality, particularly in vulnerable regions where such environmental changes cause a risk of food insecurity. In this work, we review two strategies aiming to increase drought stress tolerance: (i) the use of natural genes that have evolved over time and are preserved in crop wild relatives and landraces for drought tolerance breeding using conventional and molecular methods and (ii) exploiting the reservoir of neglected and underutilized species to identify those that are known to be more drought-tolerant than conventional staple crops while possessing other desired agronomic and nutritive characteristics, as well as introducing them into existing cropping systems to make them more resilient to water deficiency conditions. In the past, the existence of drought tolerance genes in crop wild relatives and landraces was either unknown or difficult to exploit using traditional breeding techniques to secure potential long-term solutions. Today, with the advances in genomics and phenomics, there are a number of new tools available that facilitate the discovery of drought resistance genes in crop wild relatives and landraces and their relatively easy transfer into advanced breeding lines, thus accelerating breeding progress and creating resilient varieties that can withstand prolonged drought periods. Among those tools are marker-assisted selection (MAS), genomic selection (GS), and targeted gene editing (clustered regularly interspaced short palindromic repeat (CRISPR) technology). The integration of these two major strategies, the advances in conventional and molecular breeding for the drought tolerance of conventional staple crops, and the introduction of drought-tolerant neglected and underutilized species into existing production systems has the potential to enhance the resilience of agricultural production under conditions of water scarcity.

## 1. Introduction

Crops are dependent on rainfall, and so water scarcity is the primary productivity constraint in arid and semiarid tropical areas [1]. In these areas, water deficiency can last for periods longer than four months. Additionally, when El Niño Southern Oscillation (ENSO) occurs, the amount of water available during the rainy season significantly drops, while rainfall is concentrated within a period of a few months. As a meteorological event, drought is a period in which the potential evaporation exceeds the rainfall. Agricultural drought is the result of water flow imbalance between the environmental demands of evapotranspiration and water transport in the soil-root system [2]. In this context, drought tolerance is described as the ability of a plant to live, grow, and reproduce successfully with a limited water supply or during periodic conditions of water deficit [3]. Regarding crops, a drought response is defined as a change in yield as a consequence of impaired plant development [4].

The challenge to produce under water scarcity conditions requires integrated actions and strategies to remodel the crop genetic background and the cropping systems. For it, wild relatives and landraces and neglected and underutilized species (NUS) are a rich source of genetic diversity. Thus, crop wild relatives and landraces due to their local adaptations are a vast resource of genetic diversity for developing more productive, nutritious, and resilient crop varieties [5,6,7]. In maize, landraces from dry habitats have been used successfully in breeding for water-limited environments, and wild species that are relatives of cultivated crops have been on the agenda as possible donors for drought tolerance [8]. Similarly, several NUS are drought and heat stress-tolerant, resistant to pest and diseases, and adapted to semi-arid and arid environments [9,10,11]. However, NUS are considered those species to which little attention is paid or that are entirely ignored by agricultural researchers, plant breeders, and policymakers [12]. Some NUS show potential to be introduced in cropping systems for crop diversification, e.g., quinoa has been accepted as an alternative crop in the northern latitudes of Europe [13]. The wider use of NUS would increase the agricultural biodiversity to buffer against crop vulnerability to water scarcity and would provide the quality of food and diverse food sources to address both food and nutritional security [14].

**Figure 1:**
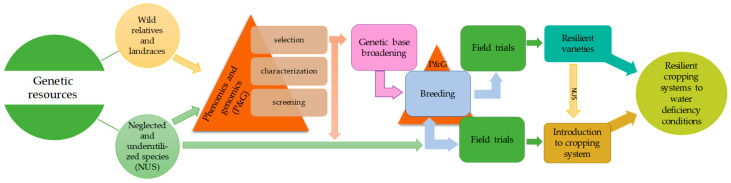
Dual strategy of breeding for drought tolerance and the introduction of underutilized crops to make more resilient cropping systems to water deficiency conditions.

This review focuses on two strategies to increase drought stress tolerance: (i) the use of natural genes that have evolved over time and are preserved in crop wild relatives and landraces and (ii) exploiting the reservoir of neglected and underutilized species to identify those that are known to be more drought-tolerant than conventional staple crops for introducing them into existing cropping systems to make them more resilient to water deficiency conditions. We also highlight the use of phenomics and genomics as methods and approaches to characterize, identify, and use the desired traits related to drought tolerance (Figure 1).

## 2. General Overview of Physiological Responses of Plants to Drought Stress Conditions

**Figure 2:**
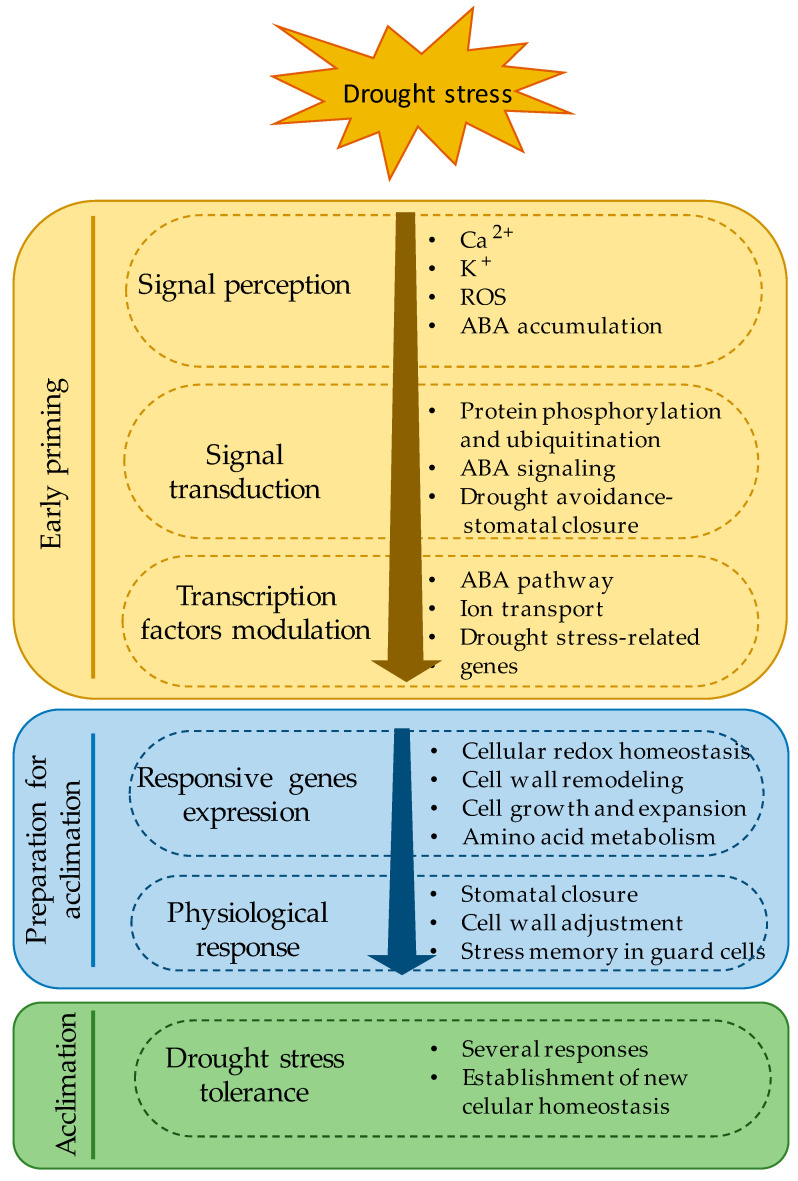
General description of physiological responses of plants to drought stress conditions. ROS: reactive oxygen species and ABA: abscisic acid.

In arid environments, crops are exposed to extreme water-limiting conditions, which have become more extreme in recent decades, leading to reductions in yield or even total yield loss. Drought conditions trigger a progressive process in plants that begins with an early priming and preconditioning stage, followed by an intermediate stage in preparation for acclimation and a late stage of new homeostasis with reduced growth (Figure 2) [15]. Signal transduction pathways connecting the recognition of environmental stress factors and the initiation of plant responses often involve several intracellular changes, including variations in Ca^2+^ concentration, reactive oxygen species (ROS) accumulation, and cytosolic K^+^. Several proteins in the plasmalemma and tonoplast recognize these intracellular messengers, acting not only during signaling sensing but, also, in response to stress conditions [16].

The cascade of morphophysiological responses to drought stress is primarily controlled by the early accumulation of abscisic acid (ABA), ion transport, and the induction of the associated signaling pathway genes [17]. Signal transduction that occurs in response to the early accumulation of ABA during drought stress is mediated by protein phosphorylation and ubiquitination. This post-translational modification of specific proteins triggers a cascade of physiological responses that include a decrease in stomatal conductance as an early avoidance response to drought stress, resulting in rapid stomatal closure [18,19,20]; furthermore, the regulation of several transcription factors (TFs) involved in osmotic stress and the increased expression of expansion genes involved in cell wall adjustments are a preparatory step towards drought acclimation [15,17,21]. TFs, as major regulators of plant responses to drought stress, affect the adaptation of plants to drought stress through their involvement in the transcriptional regulation of ABA- and drought stress-related gene expressions [21,22]. An elevated resistance to drought can also be achieved by the increased expression of cuticular wax biosynthesis genes leading to an enhanced cuticular wax accumulation in both leaves and stems [23].

ABA-dependent kinases related to stomatal closure are sucrose nonfermenting 1 (SNF1)-related protein kinase 2.6 (SnRK2.6, also known as OST1), which has the overall control of the stomata, and SnRK2.2 and SnRK2.3, which are only involved in the implementation of stress memory in guard cells during the subsequent dehydration process [24]. The OST1-dependent phosphorylation of the plasma membrane intrinsic protein 2;1 (PIP2;1) aquaporin produces an increase in the guard cell permeability to water and, possibly, hydrogen peroxide to trigger stomatal closure [25].

Drought-sensitive plants accumulate significantly more reactive oxygen species (ROS) and reactive nitrogen species (RNS) than tolerant genotypes [26,27]. Cellular redox homeostasis is disturbed as a consequence of extra ROS generation under drought stress. ABA induces the production of nitric oxide (NO) in guard cells, which, together with RNS, is a secondary messenger that modifies the enzyme activity and gene regulation [28,29]. At a specific level of NO and H_2_O_2_ treatments, the destruction of the mesophyll cell ultrastructure caused by drought stress is attenuated, increasing leaf chlorophyll, chlorophyll fluorescence values, and soluble carbohydrate and protein contents. Thus, endogenous NO and H_2_O_2_ may play crucial roles in rooting and the photosynthetic performance under drought conditions [30].

ABA is produced in the cytosol by the plastid-carotenoid pathway via the cleavage of xanthophyll precursors [20,31], and an impairment of the plastid-carotenoid pathway produces photo-oxidation and ABA-deficiency [31]. Genes involved in carotene biogenesis are not only rate-limiting for ABA synthesis but are also involved in plant responses to drought-stress conditions [20].

## 3. Use of Crop Diversity in Plant Breeding for Drought-Tolerance Traits

Valuable genes from natural inter- and intraspecific diversity can be used to take advantage of several mechanisms of survival and coadaptation in plants produced by natural selection [32]. Some of these genes are conserved by farmers (in landraces) or are present in crop wild relatives, and the narrow genetic base of modern cultivars is becoming a major bottleneck for crop improvement efforts; therefore, crop wild relatives have been extremely valuable in adapting crop varieties to changing climatic conditions [33,34]. *Helianthus anomalus*, a diploid annual sunflower species of hybrid origin that is endemic to active desert dunes, was successfully used in sunflower breeding with tolerance to drought stress [35]. In rice, *Oryza glaberrima* has been used in interspecific backcrossing to improve the drought resistance in *Oryza sativa* [36]. Similarly, the wild emmer wheat (*Triticum dicoccoides*) is highly tolerant to drought compared with its domesticated counterpart [37]. Additional examples of drought-tolerant varieties of major stables obtained through conventional breeding are presented in Table 1.

There is evidence of success in some crops that have been obtained by genetic introgression. Although, in some cases, it could be a time-demanding method, the introduction of new high-throughput “omics” technologies (some are described below) improves the efficiency for drought-tolerance traits in intra- and interspecific introgressions. In rice, a transcriptomic analysis established that drought-tolerant genotypes (drought-tolerant donor parent and progeny) were functionally enriched in oxidoreductase and lyase activities compared with a cultivated variety [61]. Thus, many traits related to mechanisms of drought tolerance have evolved over time and are present in wild relatives. The process of introducing genetic diversity from wild species into cultivars requires a significant amount of time, resources, and human capacity. The evident success of this strategy cannot be estimated only in terms of released varieties; for example, its contribution as additional genetic diversity in some crops should also be considered. However, in many species, wild relatives and landraces are poorly represented in gene banks, largely unavailable, and, therefore, underutilized [6]. For this reason, the ex situ conservation of genetic resources, especially wild relatives, should be a priority to guarantee their future availability [5,6]; however, this conservation is constrained for technical and funding challenges. Therefore, crop prioritization should be done for target wild relative conservations; ex situ conservations should be guaranteed especially for crops with importance to global food supplies and production systems worldwide [5]. Alternatively, the establishment of in situ conservation reserves could be used for major and minor crops to support the custodians of agrobiodiversity, the local communities [6]. Crops that grow in regions that currently are under high threats of water scarcity should consider seeking gene sources from wild relatives; for that, the characterizations of the wild relatives and landraces enable the detection of traits that can be used and introduced in improved varieties to provide greater adaptation and resilience to such restrictive environmental conditions.

## 4. Introduction of Neglected and Underutilized Species into Cropping Systems

The introduction of neglected and underutilized species (NUS) into the current cropping systems could help reduce food scarcity and diversify the homogeneous crops systems. This as a contribution to improve the human diet that currently has an overreliance on very limited numbers of major crops, mainly as sources of energy-dense foods but poor-quality nutrition [62,63]. As research on drought tolerance has mainly focused on major staple crops, the potential of some NUS being naturally more drought-tolerant than most staple crops has been overlooked. Moreover, NUS can be used in strategies to diversify cropping systems by introducing new species, thus increasing the genetic diversity and resilience of production systems [63]. The domestication and breeding of new crops is a long-term solution for drought constraints [64]; however, this should be considered and could be carried out through (i) understanding the physiological, genetic, and molecular basis of natural mechanisms and the plasticity that allows their adaptations to drought stress, (ii) integrating new knowledge in breeding and field crop management in priority species, and (iii) articulating strategies and actionable recommendations to encourage their cultivation and make technologies and innovations widely available [64]. Crops that are naturally adapted to arid and semi-arid regions of the world exhibit several drought-tolerance mechanisms. Halophyte plants, such as quinoa (*Chenopodium quinoa*), have adaptations to drought stress that include an increase in Na^+^ and K^+^ transporters in cell and vacuole membranes, the rapid alteration of the plasma H^+^-ATPase activity, high contents of antioxidant compounds, vesicles for salt secretion, and lower stomatal density, among others [65,66]. Although, sweet potatoes (*Ipomoea batatas*) cannot be categorized as a NUS, in certain countries, this species is not cultivated and could have high potential, since it shows the ability to grow and produce under adverse conditions. In this species, the role of phytoene synthase (IbPSY1) regulated by the orange protein (IbOr) in abiotic stress tolerance has been confirmed [67,68]. Both proteins are related to carotenoid biosynthesis and accumulation. In buckwheat, FeBREB1 and FtMYB10 are the functional genes associated with the drought response, and the proteomic profile showed an overexpression of oxidoreductase activity, oxidation–reduction processes, xyloglucan:xyloglucosyl transferase activity, and apoplasts [69,70]. Thus, some NUS grow in marginal lands and extreme conditions (drought, salinity, heat, etc.) using versatile and adaptive mechanisms.

Several NUS are hardy, resilient, and long used for food by traditional communities, particularly in the primary regions of the diversity of each crop. Although the ongoing globalization of food systems worldwide has led to a uniformization of crops grown globally at the detriment of tradition, it also has contributed to crop introductions in countries from the primary regions of diversity of the crops [71]. Therefore, countries use introduced crops from regions of diversity other than their own (“foreign crops”), confirming that crop introduction is a process that has occurred throughout the history of agricultural crops. However, this process has been affected by crop homogeneity of the global food supply, which has limited the current agriculture to a focus on eleven species [62] (these species, consequently, have been the main focus of the research activities). Thus, research focused on NUS needs to be encouraged and is required to dissect their value and promote their use as alternative crops to create more resilient cropping systems. Thanks to dedicated research, previously neglected and underutilized species such as quinoa (*Chenopodium quinoa*), buckwheat (*Fagopyrum* sp.), millet (*Pennisetum glaucum*), cowpea (*Vigna unguiculata*), and sweet potato (*Ipomoea batatas*), among others, were shown to have adaptative capacities to water deficiency and have been successfully introduced as new commercial crops into production systems (Table 2). Global efforts relating to these NUS have allowed them to be explored and introduced into agricultural systems in different regions worldwide, confirming that this is a key strategy for crop diversification, nutritional enhancement, and adaptation to changing climates for future needs.

Exploiting the potential of NUS provides a highly diversified agricultural production system capable of sustaining food and nutritional security in water-deficient environments [81]. Species such as bambara groundnut (*Vigna subterranea*), taro (*Colocasia esculenta*), teff (*Eragrostis Tef*), yam (*Dioscorea esculenta*), moringa (*Moringa oleifera*), fonio (*Digitaria exilis*), safflower (*Carthamus tinctorius*), cañahua (*Chenopodium pallidicaule*), and tepary bean (*Phaseoulus acutifolius*), among others, could be alternative crops for several regions worldwide due to their natural adaptation to arid or semi-arid regions (center of origin or diversity) [71] to contribute to crop diversification and cropping systems that are more resilient to water-deficient conditions. However, more studies should be performed to understand the natural mechanisms and plasticity that allow their adaptation to current climate alterations, to identify priority species, to design their own field crop management, and to articulate strategies and actionable recommendations to encourage their cultivation and improvement.

## 5. Methods and Approaches to Improve Crop Tolerance to Drought Stress

Several methods and approaches could be used to characterize, identify, and apply desired traits related to drought tolerance in crops and their relatives and uncover their potential to promote more resilient cropping systems. This review is focused on both phenotyping and genomic approaches.

### 5.1. Phenotyping Methods for Drought-Tolerance Trait Evaluations

Plant growth and development change because of physiological alterations in response to water deficiency. These morphological traits are related to changes in metabolic patterns in source or sink organs [82]. Morphoanatomical and physiological adaptations can be determined by measuring certain traits, primarily those related to constitutive early vigor, starch storage, growth maintenance, and desiccation tolerance. These traits are important components of crop yield but can be expressed differently among genotypes. Consequently, levels of drought tolerance are expected to differ because of the phenotypic plasticity of the genotypes. In this context, phenotyping methods could complement and improve the efficiency and accuracy of field measurements that are, subsequently, to select desirable genotypes. For example, imaging techniques are suitable to measure the response to abiotic stress [83]. A decrease in stomatal conductance produces an increase in leaf or canopy temperature, which can easily be detected by thermal imaging, and changes in the assimilation rate, stomatal conductance, and intrinsic water use efficiency can be estimated [84,85]. Hyperspectral and near-infrared imaging are also used to correlate these parameters, which can easily be monitored without destructive sampling [86]. Fluorescence imaging is used to estimate the phenotypic parameters of the photosynthetic status, quantum yield, nonphotochemical quenching, and leaf health [83].

Morphology and color distributions can serve as indicators of developmental processes and stress responses of plants, and such alterations can be effectively detected by RGB color imaging. This is an effective, quantitative, and low-cost method to determine variations in several morphological traits and other yield-related parameters, such as growth, biomass, and architecture, among others, employing routines developed to convert pixels from red/green/blue images [87]. The use of RGB imaging for plant phenotyping is improving the quality of morphoagronomic characterizations, because it uses quantitative parameters as opposed to conventional qualitative parameters. Plant growth, morphology, and physiology can be automatically monitored via nondestructive analysis using RGB imaging software that is readily available. Thus, RGB is currently the most extensive imaging technology used [88]. Additionally, traits detected by imaging can be correlated with yield, resulting in cost-effective models that can identify target traits for breeding, including drought-tolerance traits and genotypes with higher plasticity.

In roots, hydrotropism is a phenotypic strategy in response to the water supply [89]. Similar to aboveground tissues, several mechanisms are related to morphological and physiological adaptations in response to drought stress. In soybeans, the plants with more lateral roots, a thicker lateral root system, and forks showed higher yields under water-deficient conditions in clay or sandy soils compared to those of the susceptible varieties [90]. Several phenotyping systems have been developed to evaluate the response in roots to drought conditions. Those systems use high-throughput analysis to determine the stem diameter, median and maximum root width, root top, and bottom angles (using Digital Imagen of Root Traits (DIRT) [91]), properties of the primary and lateral roots (employing RootGraph [92]), and other parameters. Although image analysis has improved with time, the extraction of roots from the soil produces damage that can affect the evaluation of the actual plant response. The 3D reconstruction systems that are currently under development perform nondestructive evaluations of the root system, as they do for aboveground organs, to address this problem [93].

All the previously described imaging methods could be useful tools to characterize wild species, cultivars, landraces, and orphan crops with mechanisms to tolerate drought stress conditions. The identification of these desired traits contributes to the efficient use of genotypes with higher tolerances to drought stress, expressed primarily in yields under water-deficient conditions.

### 5.2. Potential of Genomic Approaches to Improve Crop Tolerances to Drought Stress

For the development of genetic materials with drought tolerance, selection has focused on genotypes with high yield levels cultivated under dry conditions. For example, in wheat and maize, selections have been based on evaluating the plant phenotypes and physiological responses to drought stress [94]. Selection based on plant responses to drought stress is affected by low heritability, genetic interaction, environment-genotype interactions, and polygenic effects; therefore, the selection is slow, as massive phenotypic screening is required [95]. In this context, future breeding programs focusing on drought tolerance require the combination of plant breeding, genomics, statistics, experimental design, and genetic diversity management strategies. The combinations of these approaches can offer new opportunities in the genetic dissection of important quantitative traits (e.g., drought tolerance) through the identification of quantitative trait loci (QTL), the implementation of marker-assisted selection (MAS) in breeding programs, and the cloning of these QTLs/genes and their editing using genetic-engineering strategies [94,96]. All approaches are studied using data from “omics” strategies, such as genomics, transcriptomics, proteomics, and metabolomics, which include large amounts of information; therefore, bioinformatics approaches are required for its analysis; hence, the importance of this area of computation in the analyses that integrate genotypic and phenotypic information, such as QTL mapping, single-nucleotide polymorphism (SNP), and gene discovery, as well as genomic selection, among others. Currently, the generation and use of bioinformatics tools, the open or closed code source (paid programs), and the use of molecular databases such as NCBI (National Center for Biotechnology Information) allows the analysis and interpretation of this information at different levels and varying degrees of complexity [97,98].

Genetic engineering has allowed the manipulation of the plant genome to study the gene structure and the function of candidate genes [95]. However, for drought stress, this strategy has not allowed the development of drought-tolerant varieties [94,99]. For example, maize (the highest-yielding cereal crop worldwide) is susceptible to drought during the flowering period, and the materials generated with genetic modifications to improve their drought tolerance presented a reduction in their yield traits. However, the overexpression of the trehalose-6-phosphate phosphatase (TPP) gene in maize plants has allowed the identification of materials that show an increase in yield of a similar proportion to the material generated without genetic modifications cultivated in mild and severe drought conditions [100]. Nonetheless, in Arabidopsis, the genetic basis of drought tolerance has been studied, and related homologous genes and their products have been identified and manipulated using genetic engineering, which demonstrated the feasibility for agriculturally important crop species. For example, ABA is a key player in drought tolerance and avoidance, as described above (Section 2). ABA regulates stomata closure in cases of water deficiency and plant desiccation and activates stress response genes by regulating diverse transcription factors. Therefore, if the primary aim is to increase plant sensitivity to ABA, this plant hormone is one of the main targets for genetic breeding regarding plant drought tolerance [96].

Several approaches are proposed to achieve plant tolerance to drought in transgenic crops. One approach is based on the manipulation of the ABA pathway, either by downregulating or by upregulating genes and the corresponding proteins involved in ABA signaling, biosynthesis, or degradation [7]. Farnesyltransferase ERA1 participates in ABA signaling, and plants transformed with antisense ERA1 constructed under a drought-inducible promoter showed higher tolerance to drought [101]. Another approach relies on the regulation of transcriptional activities of several genes by controlling the functions of the ABA-dependent transcription factors MYB2, MYC2, CBF, and AREB or the ABA-independent transcription factors such as DREB2, DREB3, and ZHDH [96,102,103]. ABA-independent transcription factors regulate the expression of stress-responsive genes; DREBs were first identified in Arabidopsis, but their role in stress tolerance has been demonstrated in several crops (e.g., maize, wheat, rice, rye, barley, soybean, and tomato). Hence, because DREBs have universal roles in abiotic stress responses in plants [104], they are ideal candidates for the genetic manipulation of the drought response in transgenic plant lines. Finally, but not less important, some signaling pathways that include reactive oxygen species (ROS), sucrose nonfermenting 1-related protein kinase (SnRKs), mitogen-activated protein kinases (MAPKs), calcium-dependent protein kinases (CDPKs) and phosphatases are involved in the regulation of plant responses to drought [105,106,107]; these pathways are also valuable targets in the genetic engineering of drought tolerance. The experimental results of genetic manipulation in crops are positive. In maize, the increased expression of the orthologous maize transcription factor (ZmNF-YBs) increased the tolerance to drought based on the responses of various stress-related parameters, including the chlorophyll content, stomatal conductance, leaf temperature, reduced wilting, and the maintenance of photosynthesis. These adaptations contributed to higher grain yields in water-limited environments [108]. The overexpression of phytochrome-interacting factor 3 (PIF3) increases the tolerance to dehydration and salt stress, and in ZmPIFs transgenic plants, the relative water and chlorophyll contents and chlorophyll fluorescence increases, in addition to a significant increase in cell membrane stability under stress conditions [109,110].

Drought tolerance is a quantitative trait controlled by many genes, so the genetic manipulation to generate new cultivars with drought tolerance using the single genetic interventions strategy is difficult [111,112]. However, the understanding of the genetic bases of drought tolerance has increased [101], and many genes associated with this trait have been identified [113] and used in the implementation of gene-editing systems [114], gene silencing [115] and overexpression methods [116] to generate materials with possible drought tolerance [100]. Some examples of success using these strategies to produce new materials with possible drought tolerance in the main crops are presented in Table 3. As with the phenotypic selections, the genomic analysis for drought tolerance has been studied, measuring traits related to yield in dry conditions [113], because there are few reports about regions of the genome associated with specific drought-response components. Additionally, in species such as wheat, the QTLs associated with drought tolerance remain large, and their uses in breeding programs are limited [95].

Genomic analyses for drought tolerance have allowed the identification of genes, transcription factors, miRNAs, hormones, and proteins involved in this type of stress [96], including loci related to ABA pathway signaling [124], leaf senescence [125], and other drought-related traits [96]. For example, for related ABA signaling, one of these QTLs contained a candidate gene coding for 9-cis-epoxycarotenoid dioxygenase 2, which is an essential enzyme during ABA synthesis that is expressed under drought stress [124]. QTLs related to plant growth and physiological parameters in wheat under water-deficit conditions were identified from a genetic map of 3200 SNP markers and 783 loci, and strong effects of these QTLs related to drought conditions were detected, providing evidence of their potential in plant breeding. Nevertheless, low heritability values showed that these traits are highly influenced by the environment [87]. Similarly, in barley, two primary QTLs associated with drought stress and leaf senescence were identified. Water deficiency negatively affected the biomass yield, leaf color, and electron transport rate values, whereas the osmolality, free proline content, and total content of soluble sugars increased under drought stress [125]. In rice, asymmetric root growth and an increase in the root growth angle were observed upon the introduction of the DEEPER ROOTING 1 gene (DRO1), which is a quantitative trait locus in rice that controls the root growth angle; therefore, the resulting line avoided the occurrence of drought by increasing deep rooting and maintained a high yield performance under drought conditions [126]. However, drought tolerance is a highly complex trait, so the cultivar development for this target has been achieved by classical breeding [95].

Due to the interest in drought tolerance, genomic tools such as genomic selection (GS) and marker-assisted selection (MAS) are required in new breeding programs [127]. To implement MAS, first, the identification of molecular markers and genes/QTLs that explain the phenotypic variance of drought tolerance is required, and due to drought stress in plants, many changes in gene expressions are observed. For this, the identification of candidate genes that are expressed in drought-stress conditions is a major strategy, and genomic technologies such as microarray and transcriptomic analyses have been useful in the identification of these genes [101,128]. These technologies include new strategies in the identification of candidate genes associated with important agronomic traits in species with genome sequencing that have been developed as novel in-silico platforms for gene discovery and that help scientists to identify candidate genes through the knowledge available in genetic databases and public information, allowing candidate gene prioritization [129]. Although the advance of genomic technology has allowed the identification of QTLs for drought tolerance, this information has been underutilized in the generation of new cultivars with drought tolerance [113]. To improve drought tolerance, QTLs associated with this trait have been introgressed into breeding populations and selected genotypes using MAS. For example, in rice, alleles of QTLs related to drought stress were transferred into different genetic backgrounds, and their effects were identified; moreover, the phenotypic evaluations confirmed the success of MAS for this trait [130].

Many favorable alleles for drought tolerance are present in crop wild relatives; thus, new alleles from those wild species could be included in breeding programs. These alleles should come mainly from genotypes adapted to target environments and traits. For this, it is necessary to include and evaluate these materials in all breeding programs, particularly in crops that show low levels of genetic diversity due to bottlenecks caused by selection and inbreeding [131]. In crops such as wheat and barley, QTLs related to drought have been identified and introgressed from wild relative species [132], allowing the inclusion of new favorable alleles in the breeding populations. Another approach to identifying regions that explain the phenotypic variation of traits (e.g., drought tolerance) in plants is the Genome-Wide Association Study (GWAS), where it is possible to associate a specific single-nucleotide polymorphism (SNP)/gene with a phenotypic variation of any trait [133]. The GWAS combines phenotypic and genomic information to identify statistical associations in order to explain the phenotypic variations of the target trait. This approach uses all allelic variations available and the possibility to identify marker-trait associations. On the other hand, resolution mapping depends on the linkage disequilibrium (LD) extension in the mapped population. These populations can be found in gene banks, core collections, and breeding populations, allowing the analysis of all historical recombination events in the used population. Additionally, the GWAS considers the relationship between the samples and their genetic structure in order to minimize false positives [134]. For drought stress, the GWAS has been used to identity SNPs related to high-temperature tolerance and their yield effects in crops such as wheat [135], cotton [136], rice [137], and maize [138], among other species.

Considering the above, new approaches in genomics strategies such as GS combined with high-throughput phenotyping are starting to be used to achieve different important target traits; this is because this approach allowed the identification of better lines through prediction models for complex traits such as stress (e.g., drought tolerance), employing the genomic estimated breeding value (GEBV) at the individual level [95]. GS is a form of MAS but shows new characteristics for identifying promising materials in comparison to conventional MAS that uses some markers/genes previously identified, with significant effects in the genetic government of the target traits, to improve their level [95]. In recent years, the most popular technologies to identify molecular markers in a partial representation of the genome (use of enzyme restriction) are genotyping strategies based on next-generation sequencing such as genotyping by sequencing (GBS), RAD-seq, diversity arrays technology (DArT), and the complexity reduction of polymorphic sequences (CRoPS), among others [139,140].

Genomic selection implemented prediction models that allowed selecting genotypes without phenotypic evaluation. In this sense, alleles with high and low effects are analyzed; the breeding of complex traits such as drought tolerance is well-supported by this approach. GS strategies in the plant breeding of maize and barley have reduced the selection time by almost half per cycle compared to the phenotypic selection [141]. GS in plant breeding has been used to improve different important traits such as grain yield in maize [142], soybean cyst nematode resistance [143], amylase activity in barley [144], and grain yield and plant height in rice [145], among others. To this end, genomic selection offers a new method to accelerate the breeding process [146]. The use of GS to improve drought tolerance has been reported in maize [147,148] and chickpea [149], establishing a low-to-medium prediction accuracy for yield and secondary traits related to drought stress. The implementation of GS has shown that the prediction accuracy is affected by the breeding population types, training population size, the complexity of the trait, and the number of markers used. Additionally, the inclusion of marker-trait associations identified by QTL and GWAS analyses in prediction models increases the prediction accuracy [150,151]. Thus, it is necessary to continue with the study of the genetic architecture of complex traits by GWAS and QTLs analyses.

Currently, it is possible to generate new alleles in known genes through second-generation gene-editing methods, the most popular being the zinc-finger nucleases (ZFN), transcription activator-like effector nucleases (TALEN), and the clustered regularly interspaced short palindromic repeat (CRISPR)/CRISPR-associated nuclease protein (CRISPR/Cas) system [152,153]. The CRISPR/Cas9 system is a technique to edit genes that function as an endonuclease that induces double-strand breaks (DSB) at specific genome sites, followed by DNA repair, which includes, in the gene sequences, some insertions and/or deletions [154]. This system for genome editing has been used in species such as Arabidopsis, tomato, rice, maize, and wheat [154,155,156,157], among others. This gene-editing system is known to be simple to implement, has design flexibility, is low-cost, and is highly efficient [158]. The CRISPR/Cas9 system for drought stress has been used to edit the genes ARGOS8 in maize [159]; SlMAPK3 and SlNPR1 in tomato [160,161]; MIR169a and OST2 in Arabidopsis [162]; and OsDERF1, OsPMS3, OsEPSPS, OsMSH1, and OsMYB5 in rice [163], among others.

Genetic information on drought tolerance pathways in crops has proven to be a promising approach to improve crops using genetic engineering, marker-assisted selection, and genomic selection, thus demonstrating the feasibility of integrating this approach into drought tolerance challenges in cropping systems.

## 6. Concluding Remarks

In this review, we described two strategies to improve drought-stress tolerance in crops: (i) the use natural genes for drought stress tolerance that have evolved over time and are present in crop wild relatives and landraces and (ii) exploiting the potential of neglected and underutilized species and introducing them into cropping systems to make them more resilient to water deficiency conditions. For both, the richness of genetic diversity represents an invaluable reserve for breeding, crop diversification, nutritional enhancement, and adaptation to changing climates, which should be recognized and conserved for future needs. The mechanisms of the drought tolerance of crop gene pools and neglected and underutilized species guarantee food security in environments where they grow naturally and/or are cultivated. However, despite the recent studies in this field, much information remains unknown. Future studies should continue and integrate several approaches (including both phenomics and genomics) to explore, characterize, identify, and use desired traits, contributing to the development of crops and cropping systems with tolerances to drought stress. An understanding of the morphoanatomical, physiological, and genetic mechanisms involved in the responses to drought stress in crop wild relatives and neglected and underutilized species is fundamental to recognize their potential for crop breeding or crop diversification. Therefore, the integration of crop breeding supported by phenomics and genomics is key for improving the tolerance to drought stress in crops, as has been reflected in species such as corn and other mentioned species. Likewise, for neglected and underutilized species such as quinoa, whose development as a crop in recent years can be considered as the result of the integration of these strategies and introduced into existing cropping systems. This is a practical direction for future research to make more resilient cropping systems to water deficiency conditions.

## Figures and Tables

**Table 1:** Progress in the improvement of drought tolerance (DT) in major crops, number of drought-tolerant varieties or donors selected using conventional breeding, and the use of crop wild relatives as sources of drought-tolerant genes.

Crop	No. DT Varieties or Donors	Method	Wild Species as Possible Sources for DT Introgression
Maize	41	Thirty-five varieties obtained by conventional breeding ([38,39] results published in the official internet web pages of CYMMYT, KARI, and WEMA projects)	Information not available
Potato	22	Conventional breeding [40,41,42,43,44]	*Solanum juzepczukii* B., *S. cardiophyllum*, and *S. gandarillasii*, *S. tarijense* [42]
Rice	16	Conventional breeding [45,46,47]	*Oryza longistaminata*, *O. rufipogon* [48], *O. meridionalis*, and *O. nivara* [49], *O. glaberrima* [36]
Sugarcane	24	Conventional breeding [50,51,52,53]	*Saccharum spontaneum* [54], and *S. robustum* [53]
Wheat	2	Conventional breeding [55]	*Aegilops kotsehyi*, *A. variabilis*, *A. speltoides*, *A. umbellulata*, *A. squarrosa* [54] and *A. tauschii* [56]
Cassava	2	Conventional breeding [57]	*Manihot glaziovii* [58], *M. pseudoglaziovii*, *M. stipularis*, *M. caerulenscens* [59], *M. carthaginensis*, and *M. dichotoma* [60]

**Table 2:** Neglected and underutilized species successfully introduced to several countries as alternative crops for drought tolerance.

Crop	Crop Origin	Status	Countries with Registered Varieties
Quinoa (*Chenopodium quinoa* Willd.)	Andean region [72]	Introduced	The Netherlands (5), Denmark (4), France (1), Canada(4) USA (3), Australia (2), Germany (1), Ukraine (2) [73,74]
Amaranth (*Amaranthus hypochondriacus*, *Amaranthus cruentus* L., and *Amaranthus caudatus* L.)	High tropical and subtropical lands of America [75]	Introduced	Russian (7 *Ah*, 8 *Ac*, 6 *Acr*), Germany (1 *Ah*, 1 *Acr*), Slovakia (1 *Ac*, 2 *Acr*), The Netherlands (1 *Ac*), Romania (1 *Ac*, 1 *Acr*), Ukraine (1 *Ac*), Poland (2 *Acr*), New Zealand (1 *Acr*), Czech Republic (1 *Acr*) [74]
Millet (*Pennisetum glaucum* L.)	Africa [76]	Introduced	Brazil (13), Russia (5), USA (3), Ukraine (1), Mexico (3), Australia (1) [74]
Buckwheat (*Fagopyrum* sp.)	China [77]	Introduced	Ukraine (19), Denmark (10), USA (4,) Moldova (3), Canada (2), Australia (1) [74]
Cowpea (*Vigna unguiculata*)	Southern Africa [78]	Introduced	Brazil (13), Australia (8), China (7), Turkey (7), Moldova (6), Korea (4), Romania (3), Bulgaria (2), Poland (1), Portugal (1) [74]
Sweetpotato (*Ipomoea batatas*)	Central America and north of South America [79]	Introduced	Switzerland (3), Israel (11,) Romania (2), Slovenia (8), Ukraine (4), USA (89), South Africa (29), China (42) [74]
Andean Lupin (*Lupinus mutabilis*)	Andean region [80]	Introduced	The Netherlands (1), Czech Republic (1), Germany (1) [74]

In parentheses is the number of registered varieties in each country. *Ah*: *Amaranthus hypochondriacus*, *Ac*: *Amaranthus caudatus*, and *Acr*: *Amaranthus cruentus*.

**Table 3:** Examples of the contribution of genomic approaches in the breeding of major crops for drought tolerance (DT).

Crop	Genotypes or Varieties Names	Method	DT Source	Reference
Maize	PH4CV-T, PH6WC-T, Chang7-2-T, and Zheng58-T	Overexpression	VPP gene	[117]
Potato	Cultivar Sante	Overexpression	STANN1 mRNA	[118]
Rice	U7, U14	Overexpression	OsOAT gene	[119]
Sugarcane	ZmRab17:AtDREB2A CA	Overexpression	AtDREB2A CA transcription factor	[120]
Wheat	Transgenic Durum Wheat cv. Maali	Overexpression	TdPIP2 gene	[121]
Cassava	South China 124 (SC124) cassava variety	Silencing	HSP90 protein	[122]
Soybean	Transgenic soybean plants	Overexpression	GmFDL19 transcription factor	[123]
